# CD44-targeted near-infrared photoimmunotherapy shows potent anti-tumor effects in an orthotopic lung cancer model

**DOI:** 10.1186/s12885-026-16652-5

**Published:** 2026-08-01

**Authors:** Hiroshi Yamasaki, Kohei Nakajima, Hideki Ujiie, Ryohei Chiba, Haruhiko Shiiya, Kazuto Ohtaka, Aki Fujiwara-Kuroda, Masato Aragaki, Kanako C. Hatanaka, Yutaka Hatanaka, Mikako Ogawa, Tatsuya Kato

**Affiliations:** 1 Department of Thoracic Surgery, Hokkaido University Hospital, N14W5, Kita-Ku, Sapporo, Hokkaido 060-8648 Japan; 2 Laboratory of Bioanalysis and Molecular Imaging, Graduate School of Pharmaceutical Sciences, Hokkaido University, Sapporo, Japan; 3 Center for Development of Advanced Diagnostics, Hokkaido University Hospital, Sapporo, Japan; 4 Present Address: Division of Thoracic Surgery, Department of Surgery, Faculty of Medicine, Kindai University, Osaka, Japan

**Keywords:** CD44, Lung cancer, Near-infrared photoimmunotherapy, Orthotopic lung cancer model

## Abstract

**Background:**

Lung cancer continues to be associated with a dismal prognosis, despite the recent development of multiple therapeutic modalities. Consequently, there remains an urgent need for the advancement of novel and more efficacious treatment strategies. Cluster of Differentiation 44 (CD44), a cell surface marker associated with cancer stem cells, has been implicated in tumor progression, metastasis, and resistance to therapy, thereby representing a compelling target for oncologic intervention. Near-infrared photoimmunotherapy (NIR-PIT) is an emerging cancer treatment modality that integrates the high specificity of monoclonal antibodies with cytotoxic effects induced by a photosensitizer upon exposure to NIR light. In the present study, we evaluated the therapeutic efficacy of CD44-targeted NIR-PIT utilizing an orthotopic lung cancer mouse model established via transtracheal tumor cell injection, which more faithfully recapitulates the tumor microenvironment compared to conventional subcutaneous models.

**Methods:**

An antibody–photoabsorber conjugate was synthesized by conjugating an anti-CD44 monoclonal antibody (IM7) with IRDye700DX (IR700). The cytotoxic effects of CD44-targeted NIR-PIT were assessed in vitro using CD44-expressing lung cancer cell lines. Therapeutic efficacy was subsequently evaluated in an orthotopic lung cancer mouse model established by transtracheal injection of tumor cells. Tumor progression was monitored by bioluminescence imaging, and survival and histopathological changes were analyzed following treatment.

**Results:**

In vitro experiments employing CD44-expressing cell lines demonstrated that CD44-targeted NIR-PIT induces cytotoxicity in a light dose-dependent manner. The in vivo therapeutic efficacy was subsequently assessed using the orthotopic lung cancer mouse model. Following CD44-targeted NIR-PIT, bioluminescence imaging revealed a marked reduction in tumor burden, accompanied by an exploratory difference in survival in a small cohort. Histological analysis further demonstrated tumor cell elimination and intratumoral hemorrhage in the treated lesions.

**Conclusions:**

CD44-targeted NIR-PIT demonstrated potent antitumor activity in an orthotopic lung cancer model. These findings support the potential clinical translation of CD44-targeted NIR-PIT as a novel therapeutic strategy for lung cancer. Further studies are warranted to optimize clinically feasible light-delivery methods and establish the long-term safety of this approach.

**Supplementary Information:**

The online version contains supplementary material available at https://doi.org/10.1186/s12885-026-16652-5.

## Background

Lung cancer remains the leading cause of cancer-related mortality worldwide [1, 2]. Early-stage and locally advanced non-small cell lung cancer (NSCLC) can be managed through a multimodal approach incorporating surgical resection, chemotherapy, and radiotherapy; however, advanced-stage disease continues to be associated with a poor prognosis. In recent years, molecularly targeted therapies and immune checkpoint inhibitors have improved clinical outcomes in selected patient populations; nevertheless, their efficacy is largely confined to specific subgroups, and many patients ultimately develop therapeutic resistance. Moreover, tumor recurrence and disease progression persist as significant clinical challenges. Accordingly, there is an urgent need to develop novel therapeutic strategies capable of overcoming these limitations and improving outcomes for patients with lung cancer.

Near-infrared photoimmunotherapy (NIR-PIT) is an emerging oncologic modality that utilizes conjugates composed of monoclonal antibodies (mAbs) and photosensitizers [3, 4]. The antibody component selectively binds to specific cell surface antigens, while the photoactivatable silica phthalocyanine dye, IR700, induces lethal damage to the cellular membrane upon exposure to NIR light. This highly selective approach elicits immunogenic cell death while sparing normal tissues, and its therapeutic efficacy has been demonstrated across a range of tumor antigen–antibody systems [5–9]. Following the completion of the first-in-human phase I/II clinical trial of NIR-PIT using IR700 conjugate RM-1929 in 2017, this therapy was subsequently approved in Japan for unresectable locally advanced or recurrent head and neck cancer. In addition, multiple clinical trials, including global phase III studies, are currently ongoing across Asia, North America, and Europe [10–13].

Cluster of Differentiation 44 (CD44) is a well-established marker of cancer stem-like cells and plays a critical role in intercellular adhesion, cell migration, and matrix–cell signaling pathways [14–16]. Elevated CD44 expression has been correlated with poor local tumor control and resistance to conventional therapies, thereby identifying it as a compelling target for antibody-based interventions [17–19]. Several preclinical studies have demonstrated the efficacy of CD44-targeted NIR-PIT in various syngeneic mouse models [20–22].

Although the efficacy of NIR-PIT in human lung tumors has not yet been fully elucidated, the lung represents a particularly favorable organ for light-based therapies, as near-infrared light can penetrate air-filled pulmonary tissue with minimal attenuation. While the lung is not a superficial organ readily accessible to conventional photodynamic approaches, its direct communication with the external environment via the airway permits endoscopic access to intrapulmonary lesions. This distinctive anatomical feature may facilitate efficient light delivery to tumor sites. Furthermore, CD44 is widely expressed in primary human lung cancers, underscoring its suitability as a therapeutic target [23–25].

However, the lung is also a highly vulnerable organ in which treatment-related edema, hemorrhage, or diffuse alveolar injury could result in severe respiratory compromise. Therefore, characterization of the safety profile of NIR-PIT within the pulmonary microenvironment is essential for clinical translation. Despite growing interest in NIR-PIT for thoracic malignancies, the effects of this therapy on normal lung tissue remain insufficiently investigated in clinically relevant orthotopic models.

In the present study, we investigated the therapeutic efficacy and pulmonary safety of CD44-targeted NIR-PIT using an orthotopic allograft mouse model of lung cancer established by transtracheal tumor cell injection.

## Methods

### Antibodies and reagents

An anti-mouse/human CD44-specific mAb, IM7, was procured from BioXCell (West Lebanon, NH, USA). A water-soluble silicon phthalocyanine derivative, IR700 NHS ester, was acquired from Rakuten Medical (Tokyo, Japan). All other chemicals employed were of analytical reagent grade.

### Synthesis of IR700-conjugated antibodies

The conjugation procedure was performed in accordance with a previously published protocol [4]. Briefly, IM7 (1 mg, 6.7 nmol/L) was incubated with IR700 NHS ester (65.1 μg, 33.3 nmol) in 0.1 M Na₂HPO₄ buffer (pH 8.5) at room temperature for 3 h. The resulting mixture was subsequently purified via gel-filtration chromatography using a Sephadex G-50 column (PD-10; Cytiva, Tokyo, Japan), yielding anti-CD44–IR700 conjugates.

Protein concentrations were quantified using a Modified Lowry Protein Assay Kit (Thermo Fisher Scientific, Waltham, MA, USA), with absorbance measured at 750 nm using a microplate reader (Infinite M200; Tecan Austria GmbH, Grödig, Austria). The concentration of IR700 was determined spectrophotometrically by measuring absorbance at 689 nm (UV-1800; Shimadzu Corporation, Kyoto, Japan), thereby enabling estimation of the fluorophore-to-antibody ratio. The molar ratio of IR700 to mAb was approximately 3.

### Cell lines and cell culture

Two CD44-expressing cell lines, LL/2-luc2 and AB22, were employed in this study [21, 26]. LL/2-luc2 cells (murine lung carcinoma stably expressing luciferase) were obtained from ATCC (American Type Culture Collection, Manassas, VA, USA) and maintained in high-glucose Dulbecco's Modified Eagle Medium (DMEM) (Sigma-Aldrich, St. Louis, MO, USA) supplemented with 10% fetal bovine serum (Gibco, Thermo Fisher Scientific, Waltham, MA, USA) and 1% penicillin–streptomycin (Nacalai Tesque, Kyoto, Japan). AB22 cells (murine malignant mesothelioma) were procured from ECACC (European Collection of Authenticated Cell Cultures, Salisbury, UK) and cultured in Roswell Park Memorial Institute 1640 Medium (RPMI-1640 medium) (Sigma-Aldrich) supplemented with 10% fetal bovine serum (Gibco, Thermo Fisher Scientific) and 1% penicillin–streptomycin (Nacalai Tesque). Cells were cultured in tissue culture dishes under humidified conditions at 37 °C in an atmosphere containing 5% CO₂. Cell lines were routinely screened for Mycoplasma contamination using the TaKaRa PCR Mycoplasma Detection Set (Takara Bio, Shiga, Japan).

### Fluorescence microscopic observation of the binding of IR700 conjugates

LL/2-luc2 and AB22 cells were seeded into 35-mm culture dishes and incubated for 24 h at 37 °C. Subsequently, anti-CD44–IR700 was added to the culture medium at a final concentration of 5 μg/mL, followed by incubation for 1 h at 37 °C.

Cellular fluorescence was examined using a fluorescence microscope (CKX41; Evident Corporation, Tokyo, Japan). Imaging was performed with excitation wavelengths of 673–748 nm and emission wavelengths of 765–855 nm, optimized for IR700 detection. Image analysis was conducted using ImageJ version 1.54p (National Institutes of Health, Bethesda, MD, USA).

### In vitro NIR-PIT: fluorescence microscopy

Cell viability following NIR-PIT was evaluated using LIVE/DEAD staining with calcein AM and ethidium homodimer-1 (Thermo Fisher Scientific).

LL/2-luc2 and AB22 cells were seeded in 35-mm culture dishes and incubated for 24 h at 37 °C. Anti-CD44–IR700 was subsequently added to the culture medium at a final concentration of 5 μg/mL and incubated for 1 h at 37 °C, after which the cells were exposed to NIR light for 300 s (45 J/cm^2^). Fluorescence imaging was performed 30 min following the initiation of light irradiation.

Fluorescence microscopy was conducted using excitation wavelengths of 460–490 nm and a 520 nm long-pass emission filter for calcein, and excitation wavelengths of 480–550 nm with a 590 nm long-pass emission filter for ethidium homodimer-1.

### In vitro NIR-PIT: flow cytometry

LL/2-luc2 and AB22 cells (5 × 10^5^) were seeded into 35-mm culture dishes and incubated for 24 h at 37 °C. Anti-CD44–IR700 was subsequently added to the culture medium at a final concentration of 5 μg/mL. Following incubation for 1 h at 37 °C, cells were exposed to NIR irradiation using a light-emitting diode at energy doses of 2, 4, 8, 16, and 32 J/cm^2^ and at light intensity of 50 mW/cm^2^. The light intensity was measured with an optical power meter (PM121D; Thorlabs, Newton, NJ, USA).

One hour post-irradiation, cells were collected and resuspended in Phosphate-Buffered Saline (PBS). Propidium iodide (Thermo Fisher Scientific), a marker of nonviable cells, was added to the suspension, and samples were incubated for 15 min at room temperature. Flow cytometric analysis was subsequently performed to quantify propidium iodide–positive cells following NIR-PIT. Data were analyzed using Kaluza Analysis 2.1 software (Beckman Coulter, CA, USA).

### Animal and tumor model

All in vivo experiments were conducted with the approval of the Hokkaido University Animal Care Committee and in accordance with established guidelines for the care and use of laboratory animals. Female C57BL/6JmsSlc mice (6–8 weeks of age) were obtained from Japan SLC, Inc (Shizuoka, Japan). Female mice were used throughout this study because they can be group-housed with reduced aggression and stress compared with male mice, thereby minimizing variability unrelated to the experimental intervention. Throughout all procedures, animals were anesthetized with isoflurane.

For subcutaneous tumor establishment, 5 × 10⁶ LL/2-luc2 cells suspended in 100 μL of PBS were injected into the left dorsal flank. Only mice with successfully established subcutaneous tumors were included in the therapeutic experiments. When tumors reached approximately 50 mm^3^, mice were randomly assigned to the treatment or control groups. No animals were excluded after randomization. Tumor dimensions were measured using calipers, recording the maximum longitudinal diameter (length) and transverse diameter (width). Tumor volume was calculated using the formula: volume = (length × width^2^)/2. Mice were closely monitored for signs of cutaneous toxicity following treatment. For bioluminescence imaging (BLI), D-luciferin (15 mg/mL, 200 μL, FUJIFILM Wako Pure Chemical Corporation, Osaka, Japan) was administered via intraperitoneal injection, and luciferase activity was measured using the IVIS Lumina III In Vivo Imaging System (Revvity, Waltham, MA, USA).

For the orthotopic lung tumor model, 2.5 × 10⁶ LL/2-luc2 cells suspended in 50 μL of PBS were administered via intratracheal instillation. Mice were positioned supine with the head elevated at approximately 60°, secured using a rubber band affixed to the upper incisors. The tongue was gently retracted laterally with forceps to elevate the mandible and expose the glottis. Under direct visualization of the vocal cords using a × 10 magnifying lens, a guidewire (fishing line, diameter 0.370 mm) was introduced into the trachea. Subsequently, the outer sheath of a 22-gauge intravenous catheter (Venula; Top Inc., Tokyo, Japan), a length of 32 mm, was advanced over the guidewire. Tumor cell suspension was then instilled through the cannula into the lungs. Following instillation, the mouse was maintained with the head elevated at a 45° angle until full recovery before returning to the vivarium.

To confirm successful tumor engraftment, D-luciferin (15 mg/mL, 200 μL) was administered intraperitoneally, and luciferase activity was assessed. Mice lacking detectable luciferase signals or exhibiting signal distribution in both lungs or the central airway were excluded before randomization. Eligible mice were then randomly assigned to the treatment or control groups. No animals were excluded after randomization.

### In vivo NIR-PIT

#### Subcutaneous tumor model

Fourteen mice bearing LL/2-luc2 tumors on the left dorsum were randomized into two groups to evaluate the therapeutic efficacy of NIR-PIT. In the NIR-PIT group, mice received an intravenous administration of 100 μg of anti-CD44–IR700 on day 0, followed by irradiation with near-infrared laser light (MLL-III-690–800 mW; Changchun New Industries Optoelectronics Tech Co., Ltd.) for 667 s (150 mW/cm^2^, 100 J/cm^2^) on day 1. The control group received no treatment.

Luciferase activity and IR700 fluorescence imaging were performed on day 1 (prior to NIR light irradiation) and on days 2–5 using the IVIS Imaging System (fluorescence excitation: 650–660 nm; emission: 690–730 nm band-pass).

The study endpoint was defined as a tumor volume of 1000 mm^3^, at which point mice were euthanized.

#### Lung tumor model

To evaluate the efficacy of CD44-targeted NIR-PIT in a murine lung tumor model, treatment was initiated following confirmation of solitary lung tumor establishment. A total of 100 μg of anti-CD44–IR700 was administered intravenously on day 0, followed by irradiation with near-infrared laser light (MLL-III-690–800 mW) for 333 s (300 mW/cm^2^, 100 J/cm^2^) on day 1. During irradiation, the lateral thoracic skin overlying the tumor was temporarily incised to facilitate NIR light penetration to the lung tumor and subsequently restored by suturing.

Two days post-PIT, lungs from the affected side were harvested, formalin-fixed, and paraffin-embedded, followed by sectioning at the tumor site. Tissue sections were stained with hematoxylin and eosin (H&E) and examined using an BX41 microscope (Evident Corporation, Tokyo, Japan) with 10 × and 40 × objective lenses.

Furthermore, to assess the impact of CD44-targeted NIR-PIT on survival, six mice bearing LL/2-luc2 lung tumors were randomized into two groups four days after tumor cell instillation: the NIR-PIT group received 100 μg of anti-CD44–IR700 intravenously on day 4, followed by NIR laser irradiation for 400 s (500 mW/cm^2^, 200 J/cm^2^) on day 5; the control group received no treatment.

For quantitative bioluminescence analysis, regions of interest (ROIs) encompassing the entire tumor were defined, and total photon flux was calculated for each ROI. Therapeutic efficacy was evaluated by determining the relative bioluminescence activity using the ratio: (relative light units after treatment)/(relative light units before treatment). The study endpoint was defined by the onset of pronounced dyspnea and impaired mobility, at which point mice were euthanized.

Within each figure, all IVIS images are displayed using the same color scale. Different color scales were used between figures because signal intensities differed substantially between independent experiments and imaging conditions.

Imaging analysis and histopathological evaluation were performed without blinding to treatment allocation.

### Statistical analysis

Data are presented as mean ± standard error of the mean (SEM). Statistical analyses were performed using JMP® 19 software (SAS Institute Inc., Cary, NC, USA). Data distribution was assessed using the Shapiro–Wilk test where appropriate before parametric analyses. Multiple comparisons were conducted using Dunnett’s test, while comparisons between two groups of continuous variables were evaluated using the unpaired Student’s *t*-test. Survival analysis used Kaplan–Meier estimates, and differences were tested by log-rank. A *P* value < 0.05 was considered statistically significant.

## Results

### In vitro CD44-targeted NIR-PIT

**Fig. 1:**
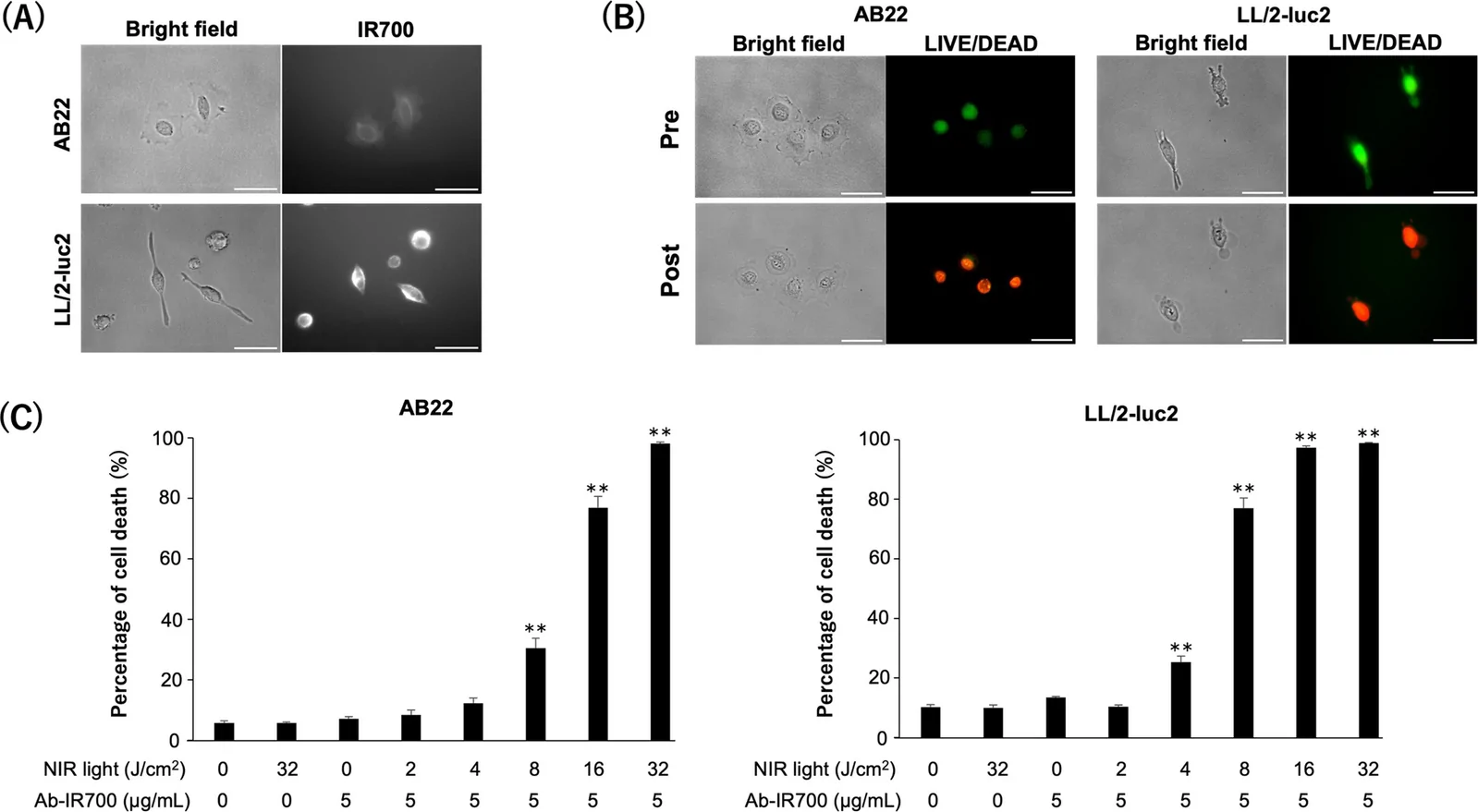
In vitro effects of CD44-targeted NIR-PIT. **A** Representative near-infrared fluorescence microscopy images showing cellular accumulation of anti-CD44–IR700 in AB22 and LL/2-luc2 cells 1 h after incubation. These images were used for qualitative confirmation of anti-CD44–IR700 accumulation. Scale bars = 50 μm (original magnification, × 40). **B** Representative fluorescence microscopy images of NIR-PIT in AB22 and LL/2-luc2 cells using the anti-CD44–IR700 conjugate. Following near-infrared irradiation, cellular swelling was observed in both cell lines. In addition, marked uptake of ethidium homodimer-1 (EthD-1; red) and leakage of calcein AM (green) were detected after NIR-PIT. Scale bars = 50 μm (original magnification, × 40). **C** Quantification of cell death induced by NIR-PIT. Flow cytometric analysis using propidium iodide staining demonstrated that cell death in both AB22 and LL/2-luc2 cells increased in a light dose-dependent manner. Data are presented as mean ± SEM (n = 5, *P < 0.05, **P < 0.01 vs. untreated control; Dunnett’s test)

Fluorescence microscopy revealed prominent IR700 signals in LL/2-luc2 and AB22 cells, indicating surface expression of CD44 and specific binding of anti-CD44–IR700 to these cells (Fig. 1A). Figure 1B presents representative microscopy images of CD44-expressing LL/2-luc2 and AB22 cells following NIR-PIT treatment. LIVE/DEAD staining demonstrated the loss of intracellular calcein and uptake of ethidium homodimer-1 after light irradiation, consistent with compromised membrane integrity. Morphological alterations, including cellular swelling and membrane blebbing—hallmarks of necrotic cell death—were also observed under bright-field imaging.

Quantitative assessment by flow cytometry revealed a light dose-dependent increase in cell death in both LL/2-luc2 and AB22 cell lines (Fig. 1C).

Collectively, these findings indicate that NIR-PIT effectively induces cytotoxicity in CD44-expressing LL/2-luc2 and AB22 cells by targeting CD44 as the antigenic determinant.

### In vivo CD44-targeted NIR-PIT

#### Subcutaneous tumor model

**Fig. 2:**
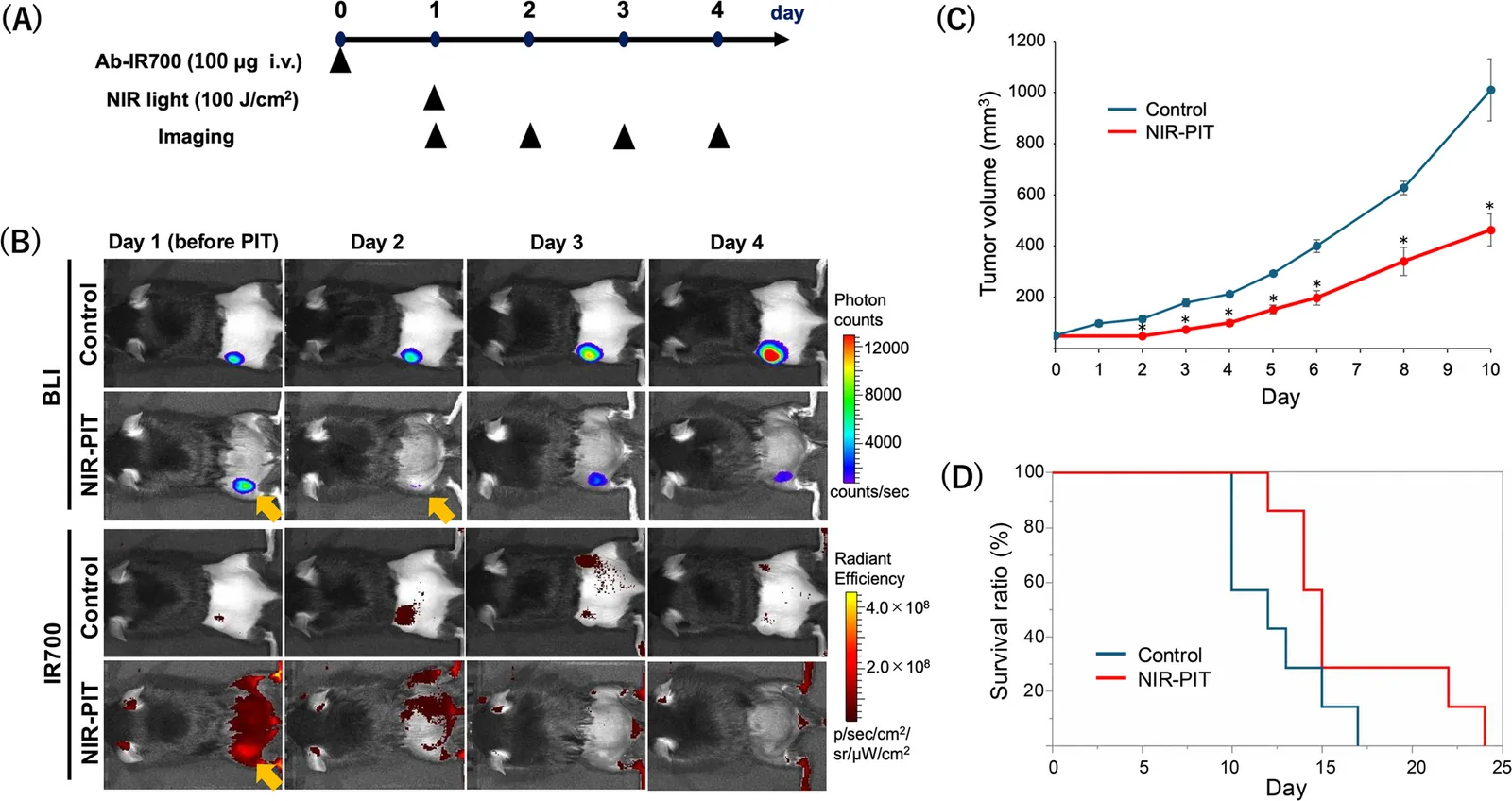
In vivo therapeutic effects of CD44-targeted NIR-PIT in a subcutaneous LL/2-luc2 tumor model. **A** Schematic illustration of the treatment protocol. Bioluminescence imaging was performed at the indicated time points. **B** Representative in vivo bioluminescence and IR700 fluorescence images of tumor-bearing mice before and after NIR-PIT at the indicated time points. **C** Tumor growth curves in the control and NIR-PIT groups. Significant suppression of tumor growth was observed in the NIR-PIT-treated group compared with the control group. Data are presented as mean ± SEM (*n* = 7, *P < 0.05 vs. control group). **D** Survival was analyzed using the Kaplan–Meier method and compared using the log-rank test

To evaluate the therapeutic efficacy of CD44-targeted NIR-PIT in lung tumors, in vivo experiments were conducted using LL/2-luc2 tumor-bearing mice. Treatment protocols and imaging schedules for the subcutaneous tumor model are illustrated in Fig. 2A. Representative serial in vivo bioluminescence and IR700 fluorescence images are shown in Fig. 2B. In the control group, bioluminescence signals progressively increased, reflecting continued tumor growth. In contrast, the NIR-PIT group exhibited a marked reduction in bioluminescence one day after treatment, consistent with rapid tumor cell death. Notably, strong IR700 fluorescence was observed in LL/2-luc2 tumors one day after administration of the anti-CD44–IR700 conjugate, indicating efficient tumor accumulation. Following NIR light irradiation, IR700 fluorescence markedly decreased, presumably because destruction of tumor cells bound by the anti-CD44–IR700 conjugate resulted in loss of the associated fluorescent signal. Following NIR-PIT, the treatment group exhibited peritumoral edema, rendering tumor diameter measurements infeasible until day 2. Nevertheless, tumor growth was significantly suppressed in the NIR-PIT group compared with the control group. Compared with the control group, the NIR-PIT group exhibited significantly smaller tumor volumes from day 2 onward (day 2, *P* = 0.0017; day 3, *P* < 0.0001; day 4, *P* < 0.0001; day 5, *P* < 0.0001; day 6, *P* = 0.0003; day 8, *P* = 0.0009; and day 10, *P* = 0.003) (Fig. 2C). Survival analysis further indicated a trend toward prolonged survival in the treatment group; however, the difference did not reach statistical significance (log-rank test, *P* = 0.0801) (Fig. 2D). These findings demonstrate that CD44-targeted NIR-PIT effectively suppresses tumor growth in a subcutaneous lung tumor model.

#### Orthotopic lung tumor model

**Fig. 3:**
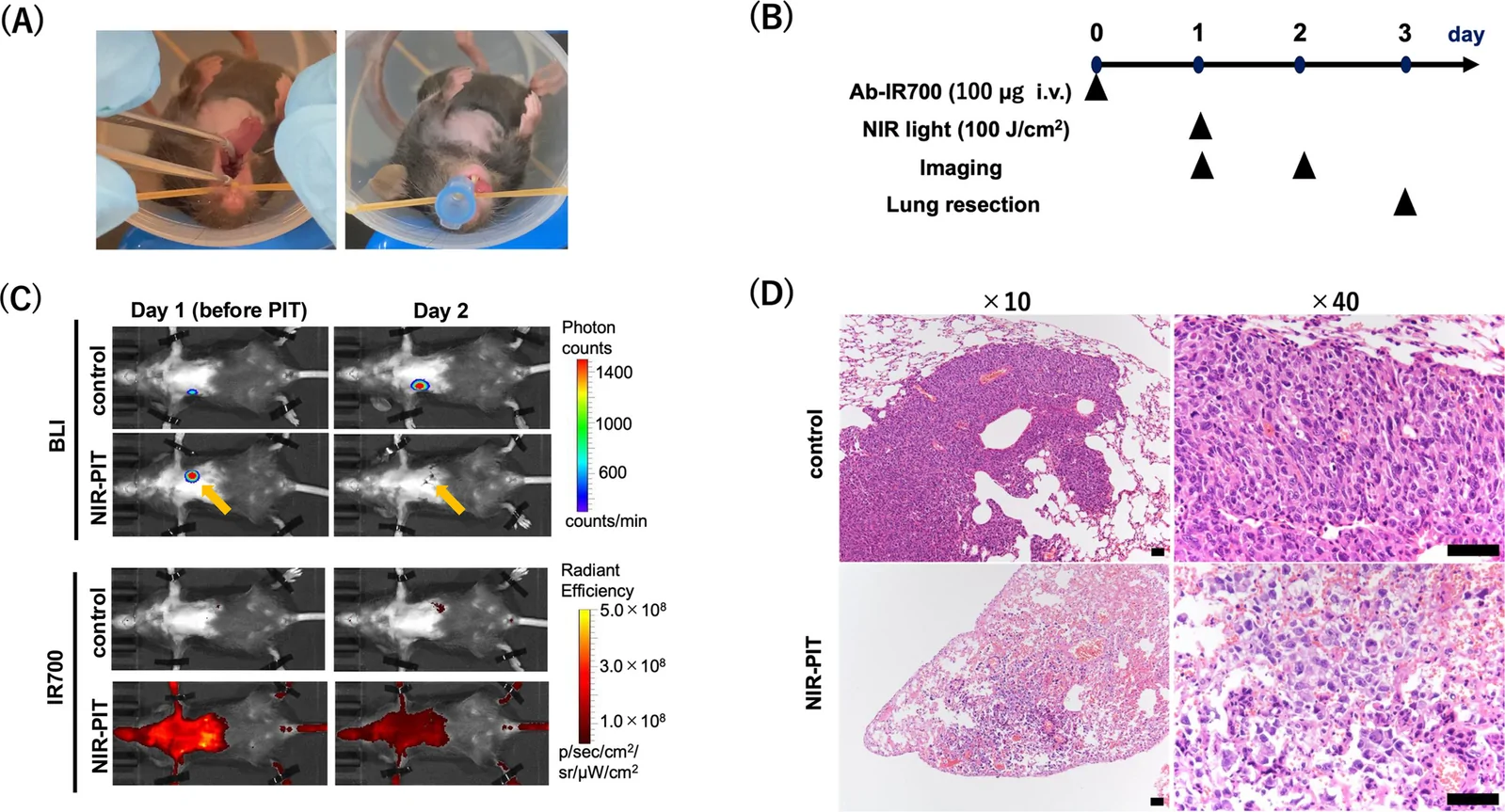
In vivo therapeutic effects of CD44-targeted NIR-PIT in an orthotopic lung cancer model. **A** Procedure for establishment of the orthotopic lung cancer model by transtracheal instillation of LL/2-luc2 cells into C57BL/6JJ mice. Tumor cell suspension was administered into the lung through a catheter inserted into the trachea under direct visualization. **B** Schematic illustration of the treatment protocol. Two days after NIR irradiation, mice were euthanized and tumor-bearing lungs were harvested for histological evaluation. **C** Representative in vivo bioluminescence and IR700 fluorescence images of tumor-bearing mice before and after NIR-PIT. **D** Representative histopathological findings of orthotopic lung tumors after treatment. The NIR-PIT group demonstrated marked therapeutic effects accompanied by microhemorrhage and extensive cancer cell death. Scale bars = 50 μm

To further assess the efficacy of CD44-targeted NIR-PIT in lung tumors, an orthotopic lung tumor model was established via transbronchial administration (Fig. 3A). In a representative case, lung extraction performed one day after anti-CD44–IR700 administration confirmed that luminescence signals were localized to a single lung, corresponding to IR700 fluorescence accumulation (Supplementary Fig. 1). NIR irradiation was subsequently conducted one day following anti-CD44–IR700 administration (Fig. 3B). In vivo bioluminescence and IR700 fluorescence images are shown in Fig. 3C. BLI demonstrated localized luminescence in LL/2-luc2 lung tumors. Bioluminescence signals increased in the control mouse, reflecting tumor growth. In contrast, the NIR-PIT-treated mouse exhibited a marked reduction in bioluminescence following treatment. Unlike the subcutaneous tumors, IR700 fluorescence appeared diffuse in the orthotopic model because fluorescence emitted from the deeply located lung tumors was attenuated and scattered by the overlying thoracic tissues. Nevertheless, ex vivo imaging demonstrated that IR700 fluorescence generally co-localized with tumor bioluminescence (Supplementary Fig. 1), supporting tumor-specific accumulation of the anti-CD44–IR700 conjugate. Two days after irradiation, mice were euthanized and tumor-bearing lungs were harvested. Histopathological evaluation using H&E staining was qualitative and intended to assess acute morphological changes. Representative histological images demonstrated localized hemorrhage, edema, and extensive tumor cell death in the NIR-PIT group (Fig. 3D). As an associated effect, localized pulmonary congestion and edema were observed in the peritumoral region, whereas no notable histological alterations were detected in the control group. Collectively, these findings indicate that NIR-PIT exerts substantial antitumor activity against LL/2-luc2 lung tumors.

**Fig. 4:**
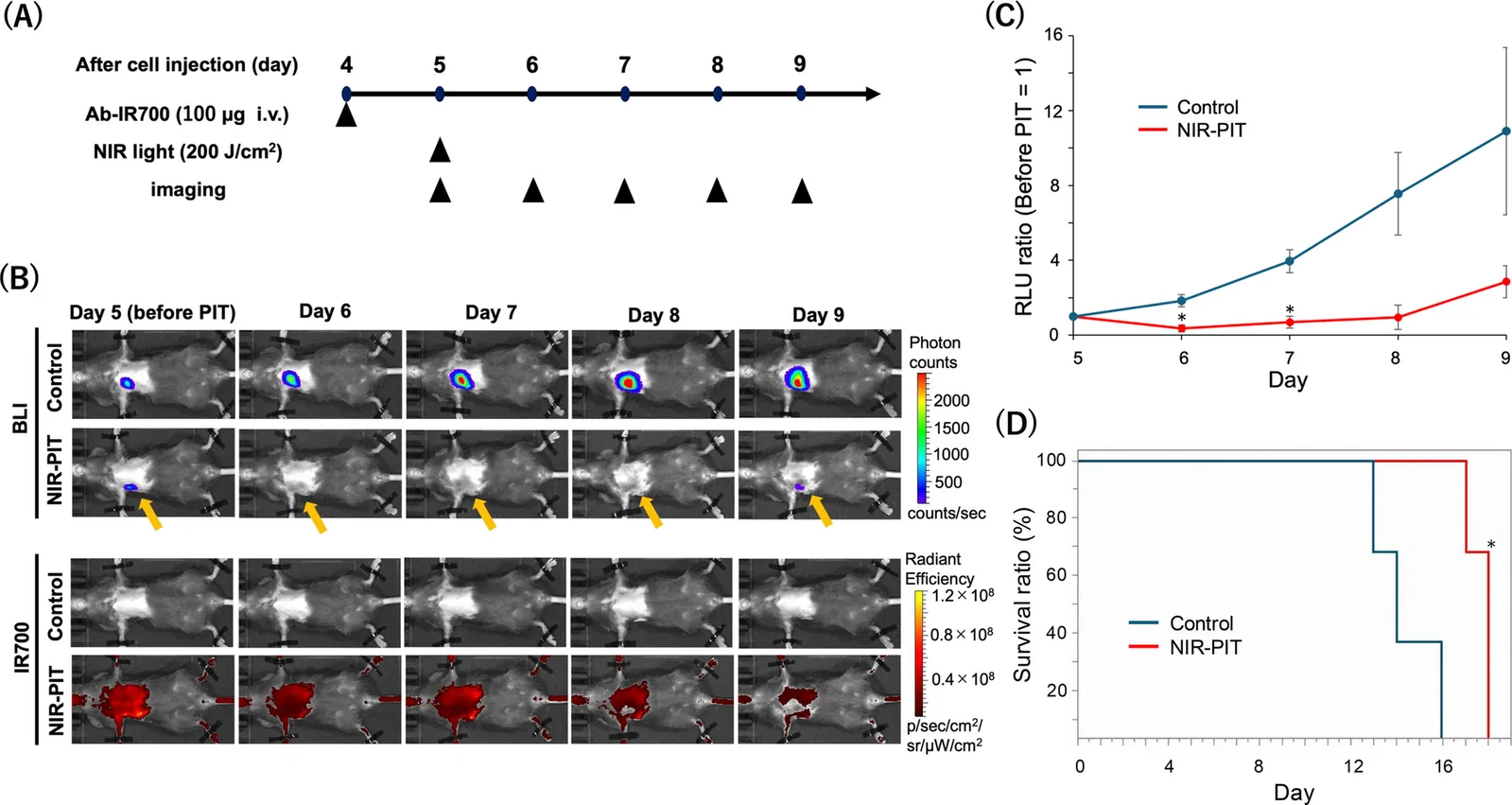
Effects of CD44-targeted NIR-PIT on survival in the orthotopic lung cancer model. **A** Schematic illustration of the treatment protocol. Bioluminescence imaging was performed at the indicated time points. **B** Representative in vivo bioluminescence and IR700 fluorescence images of tumor-bearing mice before and after NIR-PIT at the indicated time points. **C** Temporal changes in relative luciferase activity (RLU ratio) in the control and NIR-PIT groups. Significant suppression of tumor progression was observed in the NIR-PIT-treated group compared with the control group. Data are presented as mean ± SEM (*n* = 3, *P < 0.05 vs. control group). **D** Kaplan–Meier survival curves of the two groups. NIR-PIT treatment significantly prolonged survival compared with the control group (*P < 0.05)

Furthermore, the impact of CD44-targeted NIR-PIT on survival in the lung tumor model was investigated. The treatment regimen and imaging protocol are depicted in Fig. 4A, with corresponding in vivo bioluminescence and IR700 fluorescence images shown in Fig. 4B. In the control group, bioluminescence signals progressively increased, whereas the NIR-PIT group exhibited a marked reduction in bioluminescence following treatment, indicating effective tumor cell killing. Unlike the subcutaneous model, IR700 fluorescence was observed more diffusely over the thoracic region rather than as a localized tumor signal, reflecting attenuation of fluorescence from the deeper intrathoracic tumors by the overlying tissues. Quantitative ROI analysis confirmed the representative BLI findings. The ratio of bioluminescence activity after treatment to that before treatment was significantly lower in the NIR-PIT group than in the control group on days 6 and 7 (day 6, *P* = 0.0332; day 7, *P* = 0.0186) (Fig. 4C). In this exploratory analysis involving three mice per group, the survival distributions differed between the groups (log-rank *P* = 0.0246); however, this finding should be interpreted cautiously because of the small sample size (*n* = 3 per group) (Fig. 4D).

Collectively, these results suggest that CD44-targeted NIR-PIT is an effective therapeutic strategy for lung tumors, with no evidence of severe adverse effects.

## Discussion

In the present study, we demonstrated that CD44-targeted NIR-PIT exerts potent antitumor effects in an orthotopic lung cancer model while causing only limited damage to the surrounding normal lung tissue. Histopathological analyses revealed localized alveolar hemorrhage and pulmonary edema in the peritumoral region; however, these changes remained spatially confined, and no fatal pulmonary toxicity was observed. CD44 has been implicated in tumor progression, cancer stem cell maintenance, and therapeutic resistance. Accordingly, targeting CD44-expressing cells may offer a dual therapeutic advantage by not only reducing overall tumor burden but also eradicating aggressive and treatment-resistant cellular subpopulations. Although the efficacy of CD44-targeted NIR-PIT has been reported in several tumor models [20–22], this study is the first to demonstrate its therapeutic potential in an orthotopic lung cancer model.

NIR light penetrates several centimeters into biological tissue; however, achieving sufficient light delivery to lung tumors located relatively deep within the thoracic cavity remains a critical challenge [27–29]. Previous studies have demonstrated the efficacy of NIR-PIT in HER2-expressing lung metastasis models in nude mice using transcutaneous irradiation [30, 31]. In the present study, however, initial attempts employing surface illumination following hair removal failed to produce an apparent therapeutic effect. We therefore hypothesized that inadequate light penetration to the tumor represented a major limiting factor and modified the irradiation protocol by surgically removing the skin and musculature overlying the thoracic wall until the lung surface became visible. Under these conditions, a reduction in BLI signals was observed, suggesting enhanced light delivery to the tumor site.

This discrepancy may be attributable to differences in tissue thickness and optical characteristics, as C57BL/6 mice possess substantially thicker subcutaneous tissues than nude mice. Consistent with this interpretation, ex vivo evaluation of the murine thoracic wall demonstrated that NIR light transmission through the chest wall with intact skin was approximately 50% lower than that observed after skin removal (Supplementary Fig. 2). Furthermore, in certain cases, internal organs such as the heart or liver may have partially obstructed the irradiation pathway, thereby potentially influencing therapeutic efficacy.

Although limited light penetration remains a major obstacle for pulmonary NIR-PIT, advances in bronchoscopic and interstitial optical fiber systems may provide clinically feasible approaches for delivering light to deep-seated lung tumors [32, 33].

Previous studies have reported that NIR-PIT induces inflammatory responses in normal tissues surrounding the tumor, including edema [34, 35]. Therefore, we were concerned about the potential development of severe adverse events, such as extensive pulmonary edema. However, no fatal complications attributable to NIR-PIT were observed in the present study. Histopathological analyses demonstrated localized alveolar hemorrhage and pulmonary edema in the peritumoral region, but these changes remained spatially confined.

Nevertheless, several mice exhibited signs of dehydration secondary to edema within one to two days following PIT. These animals required supportive management, including adequate fluid supplementation and thermal support.

Several limitations of this study should be acknowledged. First, although CD44 expression in the cell lines used in this study has been reported previously and was qualitatively confirmed by anti-CD44–IR700 fluorescence, quantitative assessment of CD44 expression was not performed. In addition, we did not perform additional specificity controls, such as blocking experiments or isotype control conjugates, to further validate CD44-specific binding under our experimental conditions. These limitations should be considered when interpreting the targeting specificity of anti-CD44–IR700 in the present study. Nevertheless, the observed therapeutic effects were consistent with previous reports of CD44-targeted NIR-PIT.

Second, the long-term durability of the therapeutic response and the chronic adverse effects of NIR-PIT in the lung were not evaluated. In the murine model, few fatal adverse events were observed during the short-term observation period. However, long-term toxicities could not be adequately assessed because residual tumors exhibited rapid progression. Therefore, potential delayed pulmonary toxicities, including fibrosis, chronic inflammation, persistent alveolar injury, and impairment of pulmonary function, remain unknown. Long-term studies incorporating serial histopathological and functional assessments will be required to establish the chronic pulmonary safety of NIR-PIT. In addition, safety assessment was limited to acute histological changes in the lung, and systemic toxicity in other major organs was not evaluated. Therefore, the present findings do not permit comprehensive conclusions regarding the overall safety profile of CD44-targeted NIR-PIT. Future studies incorporating histological and functional evaluation of major organs will be necessary to further establish the safety of this therapeutic approach.

Third, only female mice were used. Therefore, potential sex-related differences in the therapeutic efficacy and toxicity of NIR-PIT could not be evaluated, and future studies including both sexes are warranted.

Furthermore, although previous reports have demonstrated that repeated NIR-PIT achieves superior tumor suppression [30], we evaluated only a single administration of NIR-PIT. Because NIR-PIT exerts minimal effects on surrounding normal tissues and is associated with relatively few adverse events, repeated treatment may represent a preferable therapeutic strategy. Furthermore, PIT has been reported to enhance tumor vascular permeability [36], thereby improving intratumoral drug delivery; consequently, repeated PIT exposure may further augment cancer cell death. In the orthotopic lung tumor model used in this study, the thoracic skin was surgically opened during irradiation to facilitate NIR light delivery to the lung tumor. Therefore, repeated treatment was not investigated due to concerns regarding procedural invasiveness and animal welfare. Therefore, the potential benefits of multiple treatment cycles remain to be determined.

Finally, although not investigated in the present study, synergistic effects between NIR-PIT and immunotherapies, including Programmed Death-Ligand 1 (PD-L1) inhibitors, have previously been reported [22], and similar therapeutic interactions are highly likely to occur in lung cancer. Furthermore, NIR-PIT has been shown to stimulate antitumor immune responses following treatment [37–40]. However, comprehensive evaluation of treatment-induced immune responses was beyond the scope of the present study, and the immunological mechanisms underlying CD44-targeted NIR-PIT for lung cancer remain to be fully elucidated. While numerous investigations employing orthotopic mouse models, including colorectal cancer and pleural dissemination models, have been conducted [41–47], the respiratory system—including the lungs and airways—is characterized by uniquely complex immunological environments compared with other organs [48, 49]. Consequently, NIR-PIT in lung tumors may elicit distinct immune responses, warranting further investigation.

Despite several remaining challenges, including the optimization of irradiation strategies and light-delivery systems, these findings support the potential clinical translation of CD44-targeted NIR-PIT for lung cancer, particularly in patients who are not suitable candidates for surgical resection or conventional local therapies. Furthermore, continued advances in light-delivery technology and combination strategies with immunotherapy may further enhance the clinical applicability and therapeutic efficacy of NIR-PIT for thoracic malignancies in the future.

## Conclusions

This study demonstrates the potential of CD44-targeted NIR-PIT as a local therapeutic strategy for lung cancer. Although several challenges remain for clinical translation, particularly with respect to adequate light delivery to pulmonary tumors, the rapid therapeutic effects and limited acute local toxicity observed in this study support further investigation of this approach. Further studies are required to characterize target specificity, long-term pulmonary toxicity, systemic safety, and clinically feasible light-delivery methods.

## Supplementary Information

Supplementary Material 1: Supplementary Figure 1. In vivo and ex vivo imaging of the orthotopic lung tumor model. Representative in vivo and ex vivo bioluminescence and IR700 fluorescence images of the orthotopic lung tumor model. Lung tumors showed strong bioluminescence signals, and IR700 fluorescence was observed in broadly corresponding regions.

Supplementary Material 2: Supplementary Figure 2. Evaluation of near-infrared light transmission through the murine thoracic wall. Transmitted irradiance of near-infrared light (initial intensities: 300 or 150 mW/cm²) was measured with murine chest tissues positioned 5 mm above the detector. Data are presented as mean ± SEM (*n* = 5).

## Data Availability

The datasets generated and/or analyzed during the current study are available from the corresponding author upon reasonable request.

## Abbreviations

APC: Antibody–Photoabsorber Conjugate
ATCC: American Type Culture Collection
BLI: Bioluminescence Imaging
CD44: Cluster of Differentiation 44
DMEM: Dulbecco's Modified Eagle Medium
ECACC: European Collection of Authenticated Cell Cultures
H&E: Hematoxylin and Eosin
IR700: IRDye700DX
mAb: Monoclonal Antibody
NIR: Near-Infrared
NIR-PIT: Near-Infrared Photoimmunotherapy
NSCLC: Non-Small Cell Lung Cancer
PBS: Phosphate-Buffered Saline
PD-L1: Programmed Death-Ligand 1
ROI: Region of Interest
RPMI-1640: Roswell Park Memorial Institute 1640 Medium
SEM: Standard Error of the Mean
